# New approach and new permutation tests with R programs for analyses of false-negative-contaminated data in medicine and biology

**DOI:** 10.1242/bio.045948

**Published:** 2020-01-23

**Authors:** Jaroslav Flegr, Petr Tureček

**Affiliations:** 1 Department of Philosophy and History of Science, Faculty of Science, Charles University, Viničná 7, Prague, 128 43, Czech Republic; 2 Department of Applied Neurosciences and Brain Imagination, National Institute of Mental Health, Topolová 748, Klecany, 250 67, Czech Republic

**Keywords:** Randomisation tests, Epidemiology, Serology, Case-control studies, Specificity, Sensitivity, Toxoplasma

## Abstract

Statistically, the concentration of antibodies against parasites decreases with the duration of infection. This can result in false-negative outcomes of diagnostic tests for subjects with old infections. When a property of seronegative and seropositive subjects is compared under these circumstances, the statistical tests can detect no difference between these two groups of subjects, despite the fact that they differ. When the effect of the infection has a cumulative character and subjects with older infections are affected to a greater degree, we may even get paradoxical results of the comparison – the seropositive subjects have, on average, a higher value of certain traits despite the infection having a negative effect on those traits. A permutation test for the contaminated data implemented, e.g. in the program Treept or available as a comprehensibly commented R function at https://github.com/costlysignalling/Permutation_test_for_contaminated_data, can be used to reveal and to eliminate the effect of false negatives. A Monte Carlo simulation in the program R showed that our permutation test is a conservative test – it could provide false negative, but not false positive, results if the studied population contains no false-negative subjects. A new R version of the test was expanded by skewness analysis, which helps to estimate the proportion of false-negative subjects based on the assumption of equal data skewness in groups of healthy and infected subjects. Based on the results of simulations and our experience with empirical studies we recommend the usage of a permutation test for contaminated data whenever seronegative and seropositive individuals are compared.

## Abstract (precis)

**Summary:** This article presents a new R-script for analysis of data containing a subpopulation of false-negative individuals, for example, the population of infected individuals that scored negatively in a serological assay.

## INTRODUCTION

**Fig. 1.**
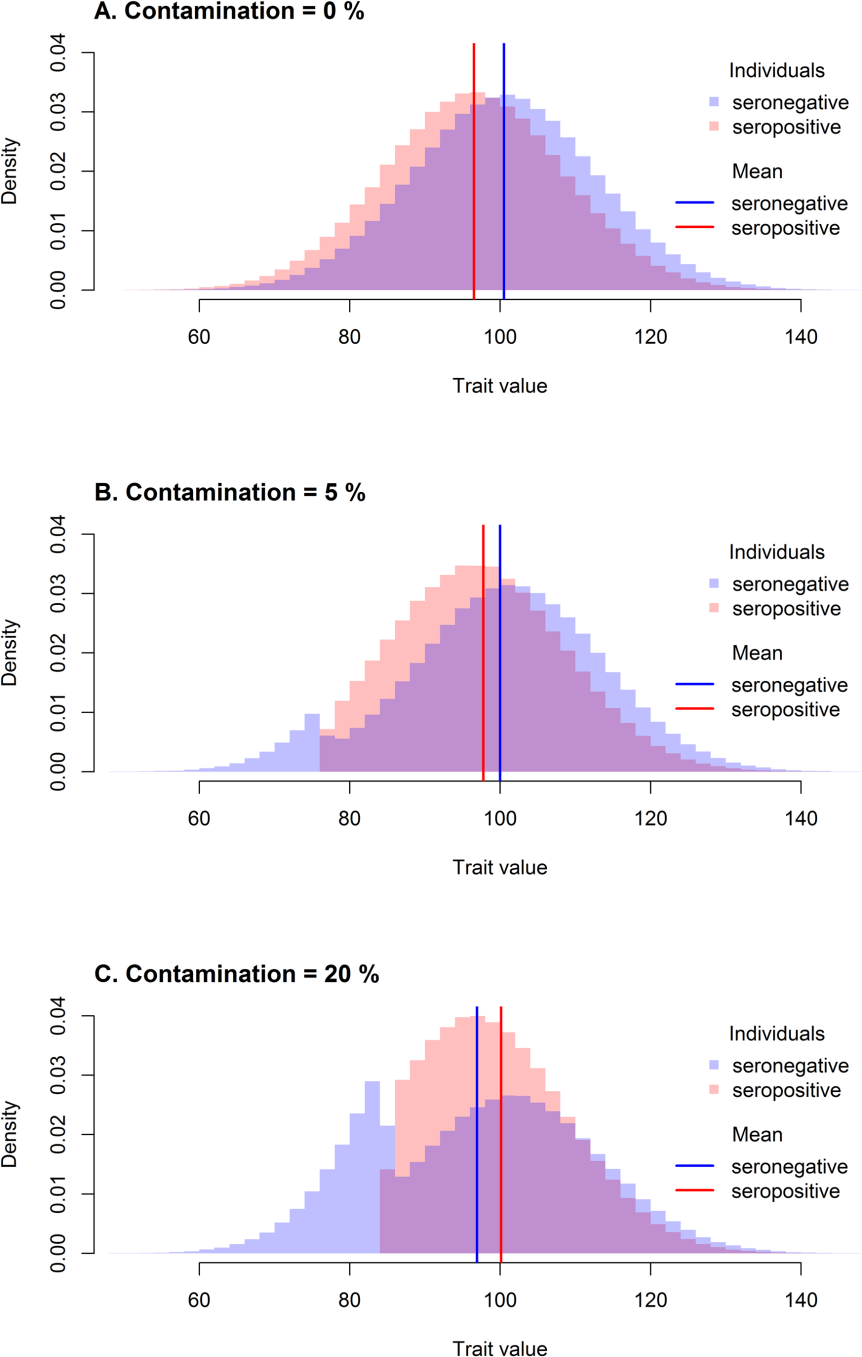
**Exemplar distributions under three different contamination levels.** The proportion of seropositive individuals (50%), the difference between healthy and infected individuals (5) and the standard deviation (10, corresponding to Cohen's d=0.5 in non-contaminated sample) within healthy individuals are held constant. Histogram C serves as a demonstration of the paradoxical result caused by a high contamination when the seronegative is a lower seropositive mean trait value despite the fact that healthy individuals score higher than infected individuals.

The reported decrease of specific antibodies measured from the onset of infection increases the risk of false-negative test results in subjects with old infections, e.g. in individuals infected in childhood (Brown et al., 1999; Celik et al., 2012; El-Sherif et al., 2012). This is also true for parasites that stay dormant in infected cells until the end of the life of infected hosts. Any subsample of seronegative subjects could, therefore, be contaminated with an unknown proportion of misdiagnosed parasite-positive individuals who became infected a long time ago (Flegr and Havlíček, 1999; Flegr et al., 2005; Griffiths et al., 1978; Chvatalova et al., 2018; Lee et al., 2008; Waner et al., 1973). This subpopulation of infected but seronegative subjects could be the most influenced by the infection (Fig. 1B) because of the long duration of their infection or because their infection took place in early stages of their ontogenesis. This could result in a paradox (Fig. 1C). The seropositive subjects could have on average higher IQ scores (or higher body weight), while the intelligence (or body weight) of seropositive subjects declines with the assessed length of infection (obtained from clinical records or assessed by the level of antibodies).

The contamination of a parasite-free subsample of false-negative individuals can be revealed and eliminated by permutation tests with the reassignment of suspect cases between subsamples (Flegr and Havlíček, 1999; Flegr et al., 2005). Such permutation tests can be performed using the program Treept, originally called PTPT (Flegr and Záboj, 1997; Flegr et al., 1998), modified for an analysis of data contaminated with an unknown number of subjects with false-negative diagnoses using the method of reassignment of potentially false-negative subjects (Flegr and Havlíček, 1999). This freeware program is available at http://web.natur.cuni.cz/flegr/treept.php. The updated version of the test suited for R can be found at https://github.com/costlysignalling/Permutation_test_for_contaminated_data in the form of comprehensibly commented R script.

Our main aim was to show that the permutation test for contaminated data does not provide false positive results, i.e. it does not return a lower *P*-value than a standard permutation test if no false-negative subjects exist in the studied population. The second aim was to develop a new tool for the skewness analysis, which can be used to estimate the approximate proportion of false-negative subjects in the studied population.

## RESULTS

**Fig. 2.**
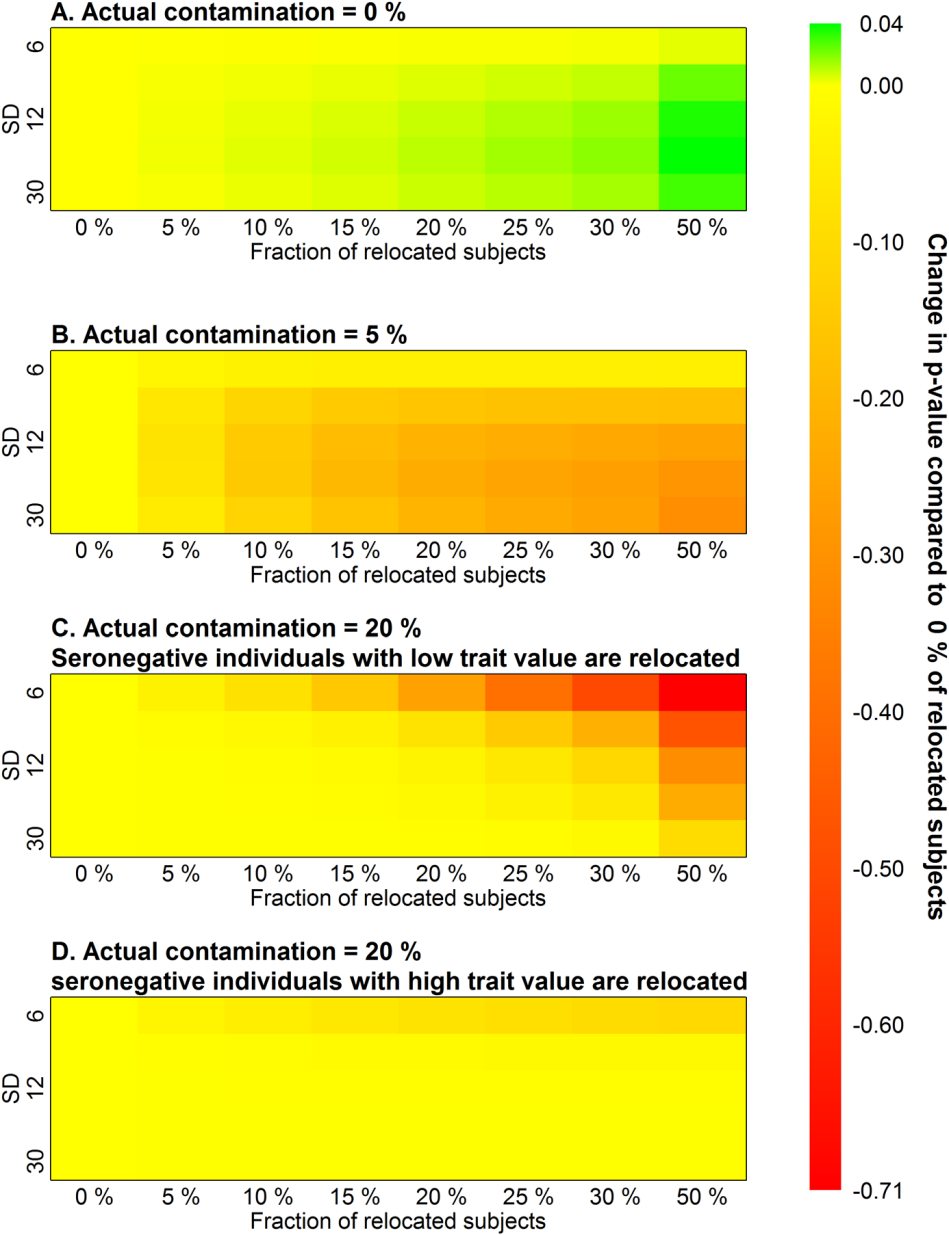
**Heatmap of the average difference between the *P*-value of standard permutation test and *P*-value of the respective permutation test for contaminated data.** One-tailed tests were used. The *P*-value increases with the fraction of relocated individuals if no actual false-negative individuals are present (A) and decreases if the sample is contaminated (B,C). This is true even if the wrong relocation direction is employed due to a paradoxical switch in the order of group means (D).

The results of the permutation analyses are shown in Table 1A. With the proportion of relocated subjects, the average *P*-value grew for every standard deviation. The visualization of this growth can be found in Fig. 2A. In this figure, the *P*-value of the standard permutation test was subtracted from each *P*-value of the permutation test for contaminated data (negative values, therefore, correspond to a decrease, and positive to an increase, of *P*-value in comparison to a standard permutation test).

**Table 1.**
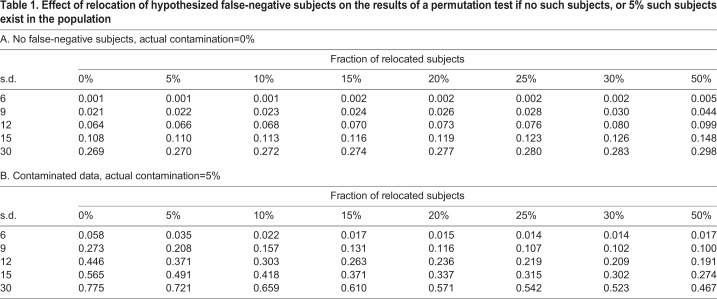
**Effect of relocation of hypothesized false-negative subjects on the results of a permutation test if no such subjects, or 5% such subjects exist in the population**

For comparison, the same computer simulation was conducted for a population of 150 seropositive and 150 seronegative individuals where 5% of seronegative individuals were false-negative individuals with extremely low intelligence (example in Fig. 1B). The average *P*-values of the permutation test for contaminated data are in Table 1B. The graphical representation of the difference between a *P*-value for 0% of relocated subjects and other contamination levels is represented in Fig. 2B, the *P*-values decrease with the proportion of relocated individuals as expected.

The table shows *P*-values computed with the permutation test for contaminated data. The simulation experiments were performed on populations that differ by variances (rows) with the relocation of different fractions of IQ-lowest individuals (columns) from the high-IQ (seronegative) group to the low-IQ (seropositive) group. *P*-values in the first column (0%, permutation test without any relocation) are lowest in their respective rows if no false-negative individuals are present (A) and highest if false-negative individuals contaminate the sample (B). For details, see the Materials and Methods section. The fixed effect was 3 IQ points. The population size was 300, and the proportion of seropositive individuals in the original sample (0% relocation) was 0.5.

Two equivalent simulations were run to demonstrate the permutation test for contaminated data on the paradoxical dataset with a high proportion of false-negative individuals. The first population of 150 seropositive and 150 seronegative individuals where 20% of seronegative subjects were false-negative individuals with extremely low intelligence (Fig. 1C). A similar one-tailed permutation test as in previous simulations was run as it was hypothesised that the average trait value of the healthy group is actually higher despite the paradoxical situation. The graphical representations of the results are in Fig. 2C. The second test with the same sample generation algorithm (150 seropositive, 150 seronegative, 20% false negative) was set to follow the default setting of the permutation test for contaminated data, which assumes the non-paradoxical situation and therefore relocates seronegative individuals with high trait value, thus widening the gap between the groups. Yielded *P*-value of one-tailed permutation test is then the proportion of random samples after relocation, where the difference between groups (seronegative–seropositive) was lower than in the original sample (Fig. 2D). In both cases, the *P*-value of the respective one-tailed permutation test decreased, so the sample with 20% false negatives was clearly distinguishable from the dataset where no false-negative subjects were present.

The appropriate direction of subject relocation can be determined on the basis of a skewness analysis of the original sample, which is available in the R version of the test at https://github.com/costlysignalling/Permutation_test_for_contaminated_data (Flegr and Turecek, 2019) if a parameter skewness.analysis is set to TRUE or as a separate function. The skewness analysis and its usage for the assessment of group mean order, as well as the contamination level estimation, is described in the Discussion. Using a two-tailed test is also worth consideration in this case. The *P*-value is then declining with the proportion of relocated individuals in all cases where false-negative individuals are present (in well-identified paradoxical situations only after the group means change their order into the right direction).

## DISCUSSION

**Fig. 3.**
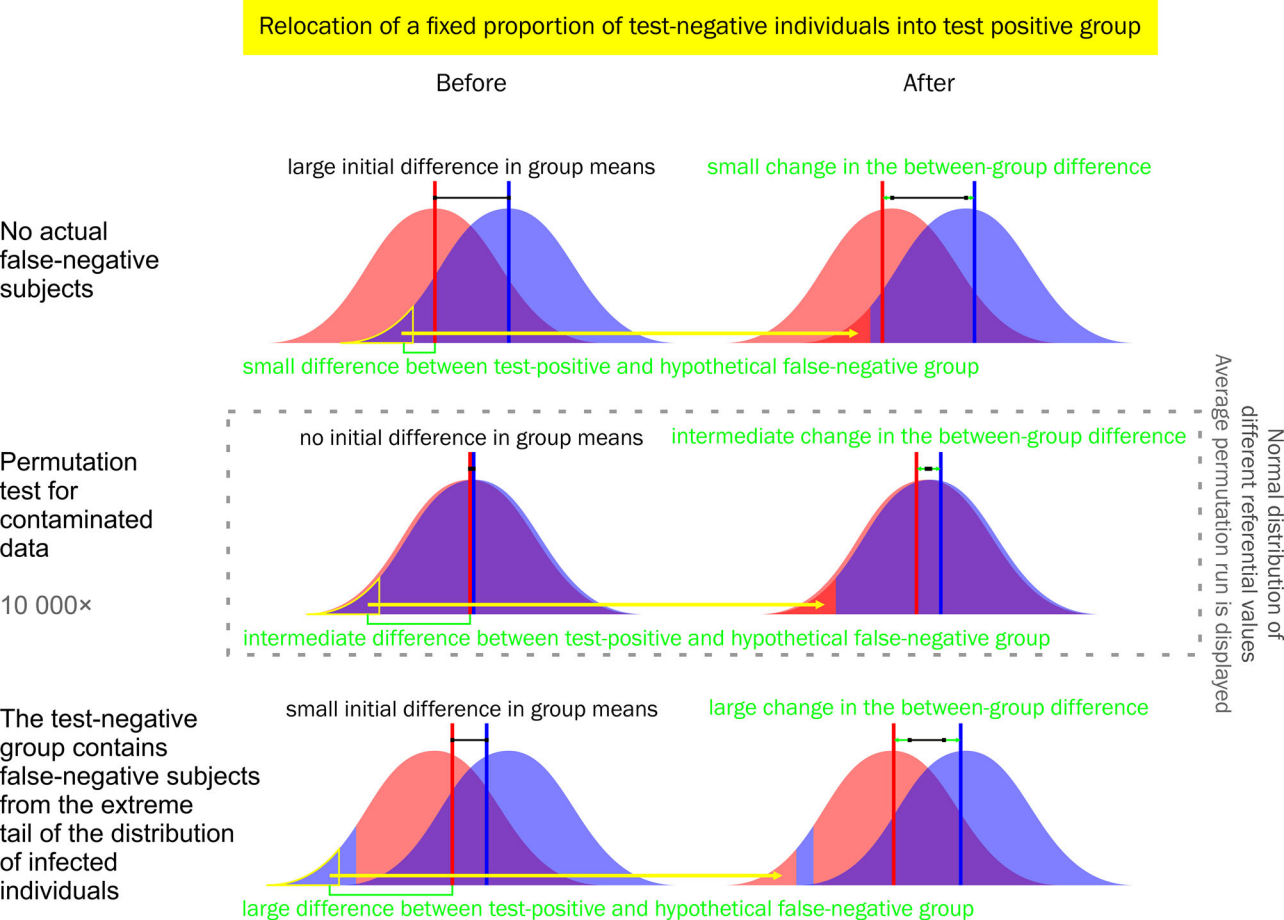
**Graphical demonstration of the intermediate position of referential permutations with relocation between empirical cases of relocation of seronegative healthy subjects and false-negative infected subjects.** The increase in *P*-value in the case of non-contaminated data is much smaller than the increase caused by possible contamination, which can completely wipe out the actual inter-group difference or even cause a paradoxical switch of the group mean order. See Table 2 or the position of 0 in the legend of Fig. 2.

The simulation results showed that the permutation test for contaminated data does not provide lower *P*-values than a standard permutation test if the experimental data does not contain a subpopulation of false-negative subjects. This test is conservative when its usage is not necessary and allows one to avoid false-negative results in the case of data contamination. This is due to the higher difference between the relocated seronegative and the original seropositive group in the presence of false-negative data. The referential set of permutations with relocation remains the same in both cases, while the relative change in inter-group difference after relocation maintains an intermediate position between those two options (see Fig. 3). Therefore, the positive result of this test, i.e. lowering the *P*-value with the growth of the proportion of relocated individuals, itself supports the hypothesis that the set of seemingly parasite-free subjects contains false-negative individuals, who, most probably, have become infected a long time ago or at a very young age. Error management clearly sides with the usage of the permutation test for contaminated data whenever the probability of false-negative subjects is not negligible (Table 2).

**Table 2.**
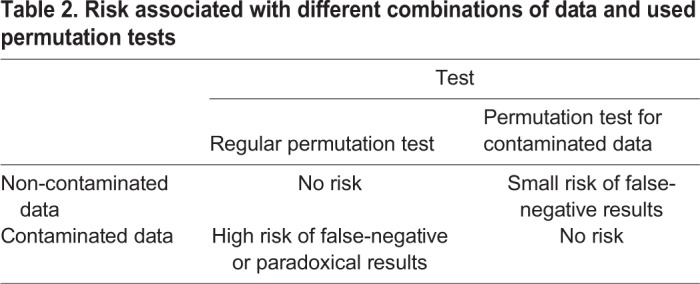
**Risk associated with different combinations of data and used permutation tests**

The pressure to avoid the high risk associated with the regular permutation test will lead us to the universal utilization of the permutation test for contaminated data whenever properties of seropositive and seronegative subjects are compared. When we conduct a skewness analysis for contaminated data prior to the permutation test, we can lower the risks further by the justification of use of the regular permutation test or an informed setting of relocated fractions of seronegative individuals in the permutation test for contaminated data.

The estimation of the actual contamination level is very difficult to discern and should be investigated more in future research. For now, we can seek assistance in a skewness analysis, which compares the skewness of trait value distribution in seropositive and seronegative groups. Skewness is defined as the third standardized moment measuring the asymmetry of the probability distribution. We assume that healthy and infected individuals have an equally skewed trait distribution. This assumption is violated if false-negative individuals are recruited from one of the extreme tails of the distribution of infected individuals. If, for example, infected individuals with the lowest trait value are identified as seronegative (as seen in Fig. 1B), the skewness of seropositive individuals becomes more positive and the skewness of seronegative individuals more negative. The exact opposite is true if individuals from the upper tail of the distribution are misdiagnosed as negative. The skewness comparison (available as a function in supplementary R script at https://github.com/costlysignalling/Permutation_test_for_contaminated_data) of contaminated data compares Fisher–Pearson coefficient of skewness of seropositive and seronegative groups under different hypothesised contamination levels and returns the skewness values for each fraction of relocated subjects, *P*-values of the difference between them based on the permutation test, the interval where group skewness is not significantly different and a proportion of relocated seronegative individuals at which the difference between group skewness was smallest (i.e. the one that generated the most similarly skewed groups). This value generally underestimates the actual contamination, but any amendments would require additional assumptions about the distribution of healthy/infected individuals, which would not necessarily be met in empirical data. Now we recommend the conduction of skewness comparison prior to the evaluation of the between-group difference and then the conduction of a permutation test for contaminated data for contamination levels between 0 and the upper border of the interval, where the difference between group skewness was not significant. We observed that the actual level of contamination in simulated data, where we can control the contamination level, rather closely matches the upper level of the similar-skewness interval due to the fact that the distributions of healthy and infected subjects largely overlap, and the extreme tail of the seronegative distribution also contains extremely healthy individuals who are relocated prior to actual false-negative individuals. For the same reason, however, we can suggest that the between-group difference for the relocated fraction where the group skewness is most similar (described above) closely matches the actual between-group difference in non-contaminated populations without false-negative subjects.

The difference between skewness coefficients in seropositive and seronegative groups in the original sample without relocated individuals can also be evaluated in the R version of the permutation test for contaminated data (set skewness.analysis to TRUE). This analysis allows one to appropriately assess whether the seronegative group includes false-negative subjects from the extreme tail of the distribution of infected individuals. By default, the permutation test for contaminated data assumes that the observed order of mean values of seropositive and seronegative groups accurately reflects the state of things in correctly determined healthy and infected groups. Therefore, the function will gradually relocate individuals from the lower tail of the distribution if the seronegative mean trait value is higher than the seropositive mean and vice versa (this can be changed by the parameter higher.healthy). If we do not alter default settings in paradoxical situations (Fig. 1C), in which the order of group means was changed due to contamination, the test algorithm will increase the difference between the groups by relocating healthy individuals from the upper tail of the distribution of seronegative subjects. The *P*-value will most likely decrease with the growing fraction of relocated individuals, as in other cases where false-negative individuals are present. This might lead to a radical misinterpretation of the data (confirmation of the assumption of higher trait value in the group of infected individuals) if attention is not paid to the skewness analysis. The skewness analysis of the original sample is not as easily fooled, since mismatching the extreme tail of infected individuals as seronegative will alter the skewness of both groups substantially. In the case of an extreme proportion of false negatives, the skewness of both groups might actually be shifted in the same direction (e.g. positive in cases similar to the example in Fig. 1C). However, the skewness of the seropositive group will be still substantially more deflected than the skewness of the seronegative group, so the skewness analysis will return reliable results.

### Conclusions

Based on the theoretical grounding described above and our experience with the research of two unrelated species of parasites, *Toxoplasma gondii* (Flegr et al., 2005, 2000) and human cytomegalovirus (Chvatalova et al., 2018), we strongly recommend the usage of permutation tests for contaminated data at https://github.com/costlysignalling/Permutation_test_for_contaminated_data whenever any properties of parasite-infected and parasite-free individuals are compared.

## MATERIALS AND METHODS

The algorithm of the one-tailed permutation test with data reassignment is as follows: the particular percentage (e.g. 5, 10, 15, 20 or 25%) of subjects with the lowest (highest) value of the dependent variable, for example IQ score, is relocated from the group of parasite-seronegative subjects to the group of the parasite-seropositive subjects. Then, the difference between the means of these two groups is calculated. In the next 10,000 steps, the empirical values of the analysed variable are arbitrarily assigned to two groups of the same size as the original seronegative and seropositive groups. The particular percentage of cases with the lowest (or highest) values of the focal variable (e.g. IQ) in the pseudo-seronegative group is relocated to the pseudo-seropositive group, and the difference between the means of the two groups is calculated. The percentage of the differences higher or equal to that calculated on the basis of the non-permuted data is considered to be the statistical significance (*P*) – the probability of obtaining the same or greater difference between the means of the two groups, if the null hypothesis is correct and the difference between the group means is as if subjects were assigned into seropositive and seronegative groups randomly.

A Monte Carlo simulation was performed with R 3.3.3. We generated a population of 150 parasite-free and 150 infected subjects (mean intelligence was 101.5 in the parasite-free group and 98.5 in the infected group – the between-group difference was 3, the population mean intelligence was 100) (Flegr et al., 2013, 2003; Chvatalova et al., 2018). Subjects were normally distributed around group means with equal standard deviations (SD). We used different SDs (6, 9, 12, 15, 30) corresponding to different effect sizes expressed by Cohen's d (0.5, 0.33, 0.25, 0.2, 0.1). Then we ran a standard permutation test. We permuted the infection status of all subjects 10,000 times and calculated a fraction of permutations where the difference between the two groups (pseudo-parasite-free and pseudo-parasite-infected subjects) was equal to or larger than the difference between the groups in non-permutated data (*P*-value of a standard permutation test). Then we repeated the analysis using a one-tailed permutation test for contaminated data. Namely, after the generation of sets of parasite-free and parasite-infected subjects (or after the generation of sets of pseudo-parasite-free and pseudo-parasite-infected subjects by permutation of the infection status), we relocated 5, 10, 15, 20, 25, 30 or 50% of subjects with the lowest intelligence from the parasite-free (or pseudo-parasite-free) set to the parasite-infected (or pseudo-parasite-infected) set. Again, we calculated a fraction of permutations with the difference between the groups equal to or greater than the value computed for the non-permuted data (*P*-values of the permutation test for contaminated data). We used populations generated for the standard permutation test (each initial population was used once for each fraction of relocated subjects). In total, 10,000 original populations were generated for each s.d.; therefore, 10,000 independent permutation tests were conducted for each combination of s.d. and each relocated fraction. The resulting *P*-values were averaged over permutation tests with the same population s.d. and the same relocated fraction.
